# Co-occurrence of thyroid and breast cancer is associated with an increased oncogenic SNP burden

**DOI:** 10.1186/s12885-021-08377-4

**Published:** 2021-06-15

**Authors:** Bence Bakos, András Kiss, Kristóf Árvai, Balázs Szili, Barbara Deák-Kocsis, Bálint Tobiás, Zsuzsanna Putz, Richárd Ármós, Bernadett Balla, János Kósa, Magdolna Dank, Zsuzsanna Valkusz, István Takács, Ádám Tabák, Péter Lakatos

**Affiliations:** 1 Department of Internal Medicine and Oncology, Semmelweis University Faculty of Medicine, 1098 Korányi S. u. 2/a, Budapest, Hungary; 2 First Department of Medicine, University of Szeged Faculty of Medicine, Szeged, Hungary; 3 Department of Epidemiology and Public Health, University College London, London, UK; 4 Department of Public Health, Semmelweis University Faculty of Medicine, Budapest, Hungary

**Keywords:** Thyroid cancer, Breast cancer, Metachronous cancer, Oncogenesis

## Abstract

**Background:**

Epidemiological evidence suggests that synchronous or metachronous presentation of breast and thyroid cancers exceeds that predicted by chance alone. The following potential explanations have been hypothesized: common environmental or hormonal factors, oncogenic effect of the treatment for the first cancer, closer follow-up of cancer survivors, shared underlying genetic risk factors. While some cases were found to be related to monogenic disorders with autosomal inheritance, the genetic background of most cases of co-occurring breast and thyroid cancer is thought to be polygenic.

**Methods:**

In this retrospective case-control study we compared the genetic profile of patients with a history of breast cancer (*n* = 15) to patients with co-occurring breast and thyroid cancer (*n* = 19) using next generation sequencing of 112 hereditary cancer risk genes. Identified variants were categorized based on their known association with breast cancer and oncogenesis in general.

**Results:**

No difference between patients with breast and double cancers was observed in clinical and pathological characteristics or the number of neutral SNPs. The unweighted and weighted number of SNPs with an established or potential association with breast cancer was significantly lower in the group with breast cancer only (mean difference − 0.58, BCa 95% CI [− 1.09, − 0.06], *p* = 0.029, and mean difference − 0.36, BCa 95% CI [− 0.70, − 0.02], *p* = 0.039, respectively). The difference was also significant when we compared the number of SNPs with potential or known association with any malignancy (mean difference − 1.19, BCa 95% CI [− 2.27, − 0.11], *p* = 0.032 for unweighted, and mean difference − 0.73, BCa 95% CI [− 1.32, − 0.14], *p* = 0.017 for weighted scores).

**Conclusion:**

Our findings are compatible with the hypothesis of genetic predisposition in the co-occurrence of breast and thyroid cancer. Further exploration of the underlying genetic mechanisms may help in the identification of patients with an elevated risk for a second cancer at the diagnosis of the first cancer.

## Background

An increased co-occurrence of breast cancer (BC) and differentiated thyroid cancer (TC) compared to chance alone has been found repeatedly in recent decades [1, 2]. Numerous studies have shown a bidirectional association between these cancers: a more pronounced risk of thyroid cancer in breast cancer survivors and a less pronounced increase in the incidence of breast cancer after thyroid cancer [2–4].

A number of hypotheses have been suggested to explain these findings [3, 4]. The one, most frequently cited, describes a common (yet unknown) hormonal etiologic factor. This hypothesis is supported by the observation of a strong female predominance of both tumor types. Estrogen has been implicated in the pathogenesis of both breast and, to a lesser degree, thyroid cancer [5]. Furthermore, thyroid dysfunction has also been tentatively linked to carcinogenesis [6–8]. It is also possible that the hormonal milieu of pregnancy (hCG, increased TRH and prolactin) would increase the thyroid and the estrogen-like effects of thyroid hormones, hence the risk of both cancer types [9]. Endocrine disrupting chemicals affecting estrogen receptors have also been theoretically implicated [10].

Another set of hypotheses emphasize carcinogenic effects of previous cancer treatment. Radio- and hormone therapy for breast cancer and I^131^ treatment for thyroid tumors have both been implicated in carcinogenesis [11–13]. It is also possible that the increased surveillance of cancer survivors could lead to earlier recognition or even overdiagnosis of a second primary malignancy.

A shared genetic background of these cancers is also a potential explanation for the observed association. Cowden and Cowden-like syndromes are characterized by the presence of hamartomas and an increased risk of, among others, both thyroid and breast cancer. These conditions are mostly monogenic disorders with an autosomal dominant inheritance. Mutations in the PTEN, SDHB, SDHD, MTHFR and PARP4 genes, and hypermethylation of KLLN have all been implicated [14–16]. Much less is known about the genetic background of sporadic cases of metachronous or synchronous breast and thyroid cancer. However, an increased familial risk of other primary malignancies of affected patients points towards underlying germline mutations [3, 17].

In the present paper, we investigated the potential for a shared genetic background of thyroid and breast cancers. The recognition and elucidation of such genetic risk factors could aid in the identification, follow-up, and potential preventive treatment of high-risk individuals with a first primary malignancy.

## Methods

### Patients and setting

We analyzed the genetic polymorphisms in 112 hereditary cancer risk genes in a case-control study of patients with a history of either breast or both breast and thyroid cancer.

Patients were selected from a pool of *n* = 274 thyroid cancer patients who had received I^131^ treatment at our institution between January 2014 and October 2018. Twenty-one of these individuals received treatment for breast cancer before or after the diagnosis of a thyroid cancer. Two patients could not have been reached for consent, resulting in a final sample of 19. Given that the risk of thyroid cancer development after breast cancer is larger than vice versa, the control group consisted of 15 subjects who received treatment for breast cancer at our institution at least 12 years before the present study. Patients with a personal or a family history of thyroid cancer were excluded from this group. These criteria were specified to minimize contamination of the control group with patients with an increased genetic risk for thyroid cancer development. No other inclusion or exclusion criteria were set.

We collected the following clinical characteristics from hospital and outpatient records: age at cancer diagnosis, tumor size, histology, staging and grading, presence or absence of lymph node and distant metastases, details of surgical-, radiation- chemotherapeutic-, hormonal and I^131^ treatment. Cancer diagnosis for all cases was based on histological data using the WHO classification of breast and endocrine tumors (4th and 3/4th edition respectively). Staging was based on the TNM system (7th edition)**.** Informed consent was obtained from all subjects before any study related procedures were performed. The study was approved by Semmelweis University Regional and Institutional Committee of Science and Research Ethics**.**

### Genetic analysis

We have aimed to cover all the well-established hereditary cancer risk loci. The final list of the 112 investigated genes was compiled based on literature data [18–20], and is shown in Table 1**.**

**Table 1:** List of cancer risk genes assessed. BC associated genes are underlined

**ACD**	**CDKN1B**	**FANCA**	**JAGN1**	**NFIX**	**PTEN**	**TERC**
**AIP**	**CDKN2A**	**FANCB**	**KIF1B**	**NHP2**	**RAD50**	**TERT**
**APC**	**CHEK2**	**FANCC**	**KIT**	**NOP10**	**RAD51**	**TINF2**
**ATM**	**DDB2**	**FANCD2**	**MAX**	**NSD1**	**RAD51C**	**TMEM127**
**ATR**	**DICER1**	**FANCE**	**MDH2**	**NTHL1**	**RAD51D**	**TP53**
**AXIN2**	**DIS3L2**	**FANCF**	**MEN1**	**PALB2**	**RB1**	**TSC1**
**BAP1**	**DKC1**	**FANCG**	**MET**	**PARN**	**RECQL**	**TSC2**
**BARD1**	**ELANE**	**FANCI**	**MITF**	**PDGFRA**	**RECQL4**	**UBE2T**
**BLM**	**EPAS1**	**FANCL**	**MLH1**	**PMS1**	**RET**	**VHL**
**BMPR1A**	**EPCAM**	**FANCM**	**MNX1**	**PMS2**	**RTEL1**	**VPS45**
**BRCA1**	**ERCC1**	**FH**	**MSH2**	**POLD1**	**SCG5**	**WAS**
**BRCA2**	**ERCC2**	**FLCN**	**MSH6**	**POLE**	**SLX4**	**WRN**
**BRIP1**	**ERCC3**	**G6PC3**	**MSR1**	**POLH**	**SMAD4**	**WT1**
**BUB1**	**ERCC4**	**GFI1**	**MUTYH**	**POT1**	**SMARCA4**	**XPA**
**CDH1**	**ERCC5**	**GREM1**	**NBN**	**PRKAR1A**	**STK11**	**XPC**
**CDK4**	**ERCC6**	**HOXB13**	**NF2**	**PTCH1**	**SUFU**	**XRCC2**

Exome amplicon library was prepared using the Ion AmpliSeq Library Kit Plus combined with the Ion AmpliSeq Exome RDY kit (ThermoFisher, MA, USA). Briefly, 100 ng of genomic DNA was added to dried down, ultra-high multiplexed primer pairs (12 pools) in a 96-well plate and amplified with the following PCR conditions: at 99 °C for 2 min; at 99 °C for 15 s and at 60 °C for 16 min (10 cycles) and holding at 10 °C. Primers were partially digested using a FuPa reagent, and then sequencing adapters and barcodes were ligated to the amplicons. The library was purified using the Agencourt AMPure XP Reagent (Beckmann Coulter, CA, USA). The concentration of the final library was determined by Ion Library TaqMan Quantitation Kit (ThermoFisher, MA, USA) on an ABI 7500 qPCR instrument with absolute quantification method.

Template preparation was performed with Ion 540 OT2 Kit (ThermoFisher, MA, USA) on semi-automated Ion OneTouch 2 instrument using emPCR method. After breaking the emulsion, the non-templated beads were removed from the solution during the semi-automated enrichment process on Ion OneTouch ES (ThermoFisher, MA, USA) machine. Later adding the sequencing primer and polymerase, the fully prepared Ion Sphere Particles (ISPs) were loaded into an Ion 540 chip, and the sequencing runs were performed using the Ion S5 Sequencing kit (ThermoFisher, MA, USA) with 500 flows.

Sequence data from the Ion Torrent run were analyzed using the platform-specific pipeline software Torrent Suite v5.10 for base calling, trim adapter and primer sequences, filtering out poor quality reads, and de-multiplex the reads according to the barcode sequences. Briefly, TMAP algorithm was used to align the reads to the hg19 human reference genome, and then, the variant caller plug-in was executed to search for germline variants in the targeted regions. Integrative genomics viewer (IGV) was used for visualization of the mapped reads. Variants were annotated using the Ion Reporter (ThermoFisher, MA, USA) software. Variant interpretation was restricted to the selected genes with known connection to oncology diseases. Variants were stratified by gene association with breast cancer and by variant association with cancer (no/potential/known) based on available data from the ClinVar database at the time of writing [21]. Synonymous mutations were not excluded from the analysis.

### Statistical analysis

Descriptive data are given as means ± standard deviation (SD) for continuous data, and frequencies (percentages) for categorical variables. Between-group differences in clinical parameters were assessed using 2-sample t tests for continuous variables, and χ2 tests or Fisher exact tests for categorical variables.

When comparing the number of different variants across groups, the assumptions of normality and homogeneity of variances were assessed using the Shapiro–Wilk test and Levene’s test respectively. Given that in some cases these assumptions were not met, Welch’s t-test was used to compare the number of variants with known, potential or no association with breast cancer and with any cancer. We also used the bootstrap procedure to get robust confidence intervals and *p*-values. To increase statistical power, we lumped together variants with potential and known associations using both weighted and unweighted scores. To generate the weighted scores, we gave double weight for variants with known association compared to those variants with potential association. To correct for multiple testing we applied the Benjamini-Hochberg procedure targeting a false discovery rate < 15%. A corrected two-tailed *p*-value less than 0.05 was considered statistically significant. Effect sizes were computed as adjusted standardized mean differences (Hedge’s g). Analyses were conducted using IBM SPSS Statistics for Windows, Version 23.0 (IBM Corp. Released 2015 Armonk, New York, U.S.A.).

## Results

Table 2 displays patients’ clinical characteristics. BC only patients had a slightly younger age at the time of breast cancer diagnosis compared to the synchronous/metachronous cancer group (47.7 vs 54.4, *p* = 0.079). All other clinical parameters evaluated were similar in the BC and BC-TC groups. It is worth noting that no difference was found in the frequency of the different BC treatment modalities (irradiation, hormone or chemotherapy).

**Table 2:** Patient characteristics at BC diagnosis and at follow-up

	BC only		BC and TC		*p*-value
	Mean / No of cases	SD / %	Mean / No of cases	SD / %	
No. of patients	15		19		
Age at follow-up	67.3	9.2	62.3	10.5	0.16
**BC clinical characteristics**					
Age at BC diagnosis	47.7	10.2	54.4	11.2	0.079
BC grade					0.363
1	3	37.5%	3	20%	1
2	1	12.5%	6	40%	0.104
3	4	50%	6	40%	1
BC T stage					0.393
1	4	36.4%	7	58.3%	0.715
2	6	54.5%	5	41.7%	0.475
4	1	9.1%	0	0%	0.441
Lymph node metastasis	5	33.3%	8	42.1%	0.728
Vascular invasion	2	22.2%	2	15.4%	1
HER2-positivity	6	40%	4	21%	0.276
ER-positivity	11	73%	10	52%	0.296
PR-positivity	8	53%	8	42%	0.730
Invasive ductal carcinoma	13	86.7%	16	84.2%	1
Distant metastasis	3	20%	2	10.5%	0.634
BC radiation therapy	14	93.2%	17	89.5%	1
BC chemotherapy	10	66.7%	9	60%	1
BC hormone therapy	11	73.3%	10	62.5%	0.704
BC targeted therapy	1	6.7%	2	12.5%	1
BC relapse	4	26.7%	3	15.8%	0.672
**TC clinical characteristics**					
Age at TC diagnosis	–	–	53.5	15	–
Papillary histology	–	–	18	94.%	–
Follicular histology	–	–	1	5.3%	–
Lymph node metastasis	–	–	4	21%	–

The results of the genetic analyses for the BC-TC patients and the control group are presented in Tables 3 and 4 respectively. Details of the statistical analysis are reported in Table 5.

**Table 3:** List of any cancer (and breast cancer) related genes and unique variants identified by individual patients in the BC-TC group. The strength of known association of each variant with oncogenesis is marked as known (**), potential (*) or no (no marker)

TC-BC patients (no. of SNPs)	Gene (***known breast cancer gene***)	SNP	Effect of mutation *- potential **- known association
T + 1 (4)	POLE	c.1738C > A	p.His580Asn*
	BUB1	c.677C > T	p.Ala226Val*
	WRN	c.355 + 4G > C	intronic*
	ERCC2	c.545C > T	p.Ala182Val*
T + 2 (4)	SMARCA4	c.918G > C	p.Gln306His*
	TSC1	c.3109_3110insGCA	p.Gly1037_Ser1038insSer*
	BIVM-ERCC5,ERCC5	c.1954C > G, c.592C > G	p.Pro652Ala, p.Pro198Ala*
	NBN	c.511A > G	p.Ile171Val*
T + 3 (5)	ATR	c.2924 T > C	p.Leu975Ser*
	KIT	c.2695A > G	p.Met899Val*
	BMPR1A	c.563G > A	p.Arg188His*
	***PALB2***	c.522A > G	p.(=)*
	HOXB13	c.251G > A	p.Gly84Glu**
T + 4 (3)	***BRCA1***	c.3925A > C	p.Asn1309His*
	CDKN2A	c.-15_8delGGCGGCGGGGAGCAGCATGGAGCC	p.Glu2_Pro3del**
	CDK4	c.625C > T	p.Arg209Cys
T + 5 (5)	SMAD4	c.845A > C	p.His282Pro*
	XPC	c.155C > T	p.Ser52Leu*
	VPS45	c.566A > G	p.Glu189Gly*
	FANCM	c.527C > T	p.Thr176Ile
	***ATM***	c.4388 T > G	p.Phe1463Cys
T + 6 (4)	ATR	c.4357A > G	p.Ile1453Val*
	***ATM***	c.8983C > A	p.Leu2995Ile*
	***BRCA2***	c.8755-1G > A	intronic**
	***BRCA1***	c.692C > T	p.Thr231Met*
T + 7 (6)	MUTYH	c.1276C > T	p.Arg426Cys
	ATR	c.4912C > T	p.Gln1638Ter**
	ATR	c.1546A > G	p.Thr516Ala*
	NSD1	c.3805 T > C	p.Ser1269Pro*
	***BRCA2***	c.6968A > G	p.His2323Arg*
	***CHEK2***	c.614C > T	p.Thr205Ile*
T + 8 (4)	ATR	c.3424A > G	p.Ser1142Gly*
	PDGFRA	c.842C > T	p.Thr281Met*
	TINF2	c.488C > G	p.Pro163Arg*
	FLCN	c.592G > A	p.Asp198Asn
T + 9 (1)	TERT	c.1234C > T	p.His412Tyr
T + 10 (6)	TSC2	c.3820 T > C	p.Ser1274Pro*
	FANCI	c.2011A > G	p.Ile671Val
	FANCI	c.2604A > C	p.Glu868Asp
	MFSD3	c.1033C > T	p.Arg345Cys*
	***BRCA2***	c.1483G > C	p.Ala495Pro*
	***BRCA2***	c.4409_4410delTA	p.Ile1470fs**
T + 11 (2)	PTCH1	c.4324C > T	p.Arg1442Trp
	***CDH1***	c.32 T > C	p.Leu11Pro*
T + 12 (5)	PMS1	c.2783 T > C	p.Leu928Pro*
	***ATM***	c.1273G > A	p.Ala425Thr*
	***ATM***	c.1300C > T	p.Pro434Ser*
	***BRCA2***	c.9038C > T	p.Thr3013Ile
	STK11	c.413A > G	p.Glu138Gly*
T + 13 (2)	MUTYH	c.536A > G	p.Tyr179Cys**
	SLX4	c.2359G > A	p.Glu787Lys
T + 14 (3)	FANCG	c.634G > A	p.Ala212Thr*
	***CHEK2***	c.1556G > T	p.Arg519Leu*
	KIF1B	c.2680G > A	p.Val894Met*
T + 15 (2)	RECQL	c.1360C > T	p.Arg454Cys*
	***CHEK2***	c.444 + 1G > A	intronic**
T + 16 (4)	TSC1	c.2418G > A	p.Met806Ile*
	POLD1	c.835_837delGAG	p.Glu279del*
	PDGFRA	c.1099G > A	p.Val367Met*
	DIS3L2	c.1447C > G	p.Arg483Gly*
T + 17 (3)	RECQL4	c.3062G > A	p.Arg1021Gln
	SLX4	c.3890G > A	p.Gly1297Glu
	SLX4	c.179A > C	p.Gln60Pro
T + 18 (4)	MSH6	c.3226C > T	p.Arg1076Cys**
	***ATM***	c.7290 T > G	p.His2430Gln*
	MAX	c.25G > T	p.Val9Leu*
	***BRCA1***	c.181 T > G	p.Cys61Gly**
T + 19 (0)	–	–

**Table 4:** List of any cancer (and breast cancer) related genes and unique variants identified by individual patients in the control group. The strength of known association of each variant with oncogenesis is marked as known (**), potential (*) or no (no marker)

BC only patients (no. of SNPs)	Gene (***known breast cancer gene***)	SNP	Effect of mutation *- potential **- known association
T-1 (3)	***ATM***	c.8965C > G	p.Gln2989Glu*
	MUTYH	c.1435G > A	p.Glu479Lys*
	FANCB	c.2435A > G	p.Tyr812Cys*
T-2 (2)	MSR1	c.919G > T	p.Asp307Tyr*
	***ATM***	c.6067G > A	p.Gly2023Arg
T-3 (4)	RAD50	c.1741C > T	p.His581Tyr*
	MITF	c.1255G > T	p.Glu419Ter**
	WRN	c.95A > G	p.Lys32Arg
	APC	c.7490C > T	p.Ser2497Leu
T-4 (6)	ELANE	c.341 T > C	p.Leu114Ser*
	RB1	c.10A > C	p.Lys4Gln*
	WRN	c.1149G > T	p.Leu383Phe
	ERCC4	c.1135C > T	p.Pro379Ser
	MSR1	c.667 T > A	p.Ser223Thr*
	WRN	c.2983G > A	p.Ala995Thr
T-5 (1)	TSC1	c.1390G > A	p.Gly464Ser*
T-6 (1)	***CHEK2***	c.190G > A	p.Glu64Lys**
T-7 (3)	MSH2	c.2187G > A	p.Met729Ile*
	RAD50	c.734A > G	p.Glu245Gly*
	FANCA	c.2658G > C	p.Glu886Asp*
T-8 (2)	***CDH1***	c.344C > T	p.Thr115Met*
	MET	c.2962C > T	p.Arg988Cys
T-9 (6)	MDH2	c.415G > A	p.Val139Ile*
	FANCA	c.1874G > C	p.Cys625Ser
	ATR	c.7303A > G	p.Ile2435Val*
	TERT	c.2726 T > C	p.Val909Ala*
	***PALB2***	c.2483G > A	p.Cys828Tyr*
	FANCB	c.454 T > C	p.Phe152Leu*
T-10 (3)	RECQL4	c.616A > C	p.Lys206Gln*
	BLM	c.3536C > T	p.Thr1179Ile*
	FANCC	c.632C > G	p.Pro211Arg
T-11 (3)	MITF	c.1334C > A	p.Thr445Lys
	WRN	c.1211 T > C	p.Ile404Thr
	SLX4	c.179A > C	p.Gln60Pro
T-12 (1)	FANCI	c.824 T > C	p.Ile275Thr
T-13 (3)	BUB1	c.307A > G	p.Ile103Val
	FANCA	c.1874G > C	p.Cys625Ser
	AXIN2	c.1994delG	p.Gly665fs*
T-14 (3)	RECQL4	c.941C > T	p.Pro314Leu
	SLX4	c.4963A > G	p.Arg1655Gly
	RAD51C	c.130 T > C	p.Ser44Pro
T-15 (1)	PMS1	c.1609G > A	p.Glu537Lys*

**Table 5:** Comparison of the two patient groups by the number of genetic variants

	BC only		BC and TC		*p*-value
	Mean	Std. error	Mean	Std. error	
*Genes associated with BC*					
No. of SNPs with potential/ known association with BC	0.27	0.12	0.84	0.22	**0.029**
No. of SNPs with potential/ known association with BC **(weighted scores)**	0.17	0.08	0.53	0.15	**0.039**
*112 cancer risk genes*					
No. of SNPs with no known association with oncogenesis	1.20	0.30	0.74	0.20	0.206
No. of SNPs with a potential association with oncogenesis	1.40	0.39	2.32	0.37	0.096
No. of SNPs with a known association with oncogenesis	0.20	0.11	0.47	0.14	0.131
No. of SNPs with potential/ known association with oncogenesis	1.60	0.36	2.79	0.39	**0.032**
No. of SNPs with potential and known association with oncogenesis **(weighted scores)**	0.90	0.18	1.63	0.23	**0.017**

We found a significantly lower number of SNPs with potential or known association with breast cancer in the BC only group (mean difference − 0.58, BCa 95% CI [− 1.09, − 0.06], t (27) = − 2.30, *p* = 0.029, g = 0.74 for unweighted and mean difference − 0.36, BCa 95% CI [− 0.70, − 0.02], t(27.26) = − 2.17, *p* = 0.039, g = 0.70 for weighted scores).

The individual number of SNPs with no known genetic association with cancer was nominally higher in the BC only group (mean difference 0.46, BCa 95% CI [− 0.27, 1.20], t(25.56) = 1.30, *p* = 0.206, g = 0.39), while the number of potentially associated SNP or those with a known association was nominally lower in the BC only group (mean difference − 0.92, BCa 95% CI [− 2.01, 0.17], t(30.98) = − 1.72, *p* = 0.096, g = 0.59 and mean difference − 0.27, BCa 95% CI [− 0.63, 0.09], t(31.37) = − 1.55, *p* = 0.131, g = 0.51, respectively).

When we lumped together SNPs with potential or known association with carcinogenesis to increase statistical power, we found significantly higher values in the synchronous/metachronous cancer group compared to the BC only group using both unweighted (mean difference − 1.19, BCa 95% CI [− 2.27, − 0.11], t(31.88) = − 2.24, *p* = 0.032, g = 0.76) and weighted (mean difference − 0.73, BCa 95% CI [− 1.32, − 0.14], t(31.80) = − 2.51, *p* = 0.017, g = 0.83) scores.

## Discussion

In our study, we found a significantly higher burden of established and potential germline cancer risk variants among subjects with a history of metachronous thyroid and breast cancer compared to patients with a history of breast cancer only. The trend was similar and effect sizes were still considerable when we assessed the number of SNPs with known and potential cancer risk separately. However, in these cases, probably due to lack of power, statistical significance was not reached. Our results are compatible with the hypothesis that the sporadic co-occurrence of thyroid and breast cancer is of multigenetic origin and probably related to the burden of the carcinogenic SNPs rather than an individual gene alteration.

Comparison of clinical features of breast cancer, such as onset, stage, histology and treatment also yielded no significant differences. There was a non-significant tendency for later age of breast cancer onset in the study group which is contrary to previous findings [22].

The metachronous and synchronous occurrence of breast and thyroid cancer has been first described nearly four decades ago [1]. The relationship between the two tumor types has since been found to be bidirectional. In recent meta-analyses, the odds ratio for thyroid cancer following breast cancer treatment was 1.55 while the odds ratio of developing BC after TC was reported between 1.18–1.32 [3, 4]. Studies exploring the cause behind this association emphasize either the role of common underlying hormonal and environmental factors, or the role of the first malignancy in the development of the second. The potential role of cancer treatment and the increased surveillance of cancer survivors fall in this latter category. With breast cancer being the most common type of malignancy in women [23] and with the increasing incidence of thyroid cancer worldwide [24, 25], the importance of this topic is clear.

One potential hypothesis explaining this bidirectional relationship implicates common hormonal factors and is rooted in the overwhelming predominance of both cancer types in reproductive-aged women [4]. Estrogen, progesterone and androgen receptors have been shown to be overexpressed not only in breast cancer but also in thyroid neoplasms [26]. In vitro and animal studies also point towards the potential oncogenic effect of estrogens in thyroid cells [26, 27]. An increased TSH secretion in response to estrogens has also been suggested as a potential pathomechanism for thyroid cancer development [3], as serum TSH levels have been shown to correlate with thyroid cancer risk [28]. TSH and thyroid hormones also have been implicated in breast carcinogenesis by in vitro animal and observational studies. T3 seems to stimulate proliferation in breast cancer cells in vitro at least in part through interactions with the estrogen signalling system [29, 30]. Tumor suppressor pathways downstream of the TRβ nuclear thyroid receptor and oncogenic pathways downstream of the membrane receptor αVβ3 have been suggested to mediate the role of thyroid hormones in carcinogenesis [31–33]. While some observational studies in humans found a decreased breast cancer incidence in hypothyroid and an increased incidence in hyperthyroid patients [7, 8], other studies have failed to replicate these findings [34, 35].

Endocrine disrupting chemicals (EDC) have been linked to numerous types of hormone dependent malignancies including both thyroid and breast cancer [36, 37]. In addition to EDCs, obesity, a shared risk factor for both tumor types has also been postulated to increase the risk of synchronous/metachronous cancer development [38, 39]. Selective estrogen receptor modulators (SERMs) are widely used in breast cancer treatment. While there is some evidence that these drugs due to their partial estrogen effect increase TSH secretion and thyroid cell proliferation, their potential role in thyroid cancer development has not yet been studied [13]. The similar frequency of hormone therapy in the BC only, and the BC-TC groups in our study point toward a limited role of sex hormones in the development of co-occurring breast and thyroid cancer.

Other theories suggest the role of prior cancer treatment in the development of the second primary malignancy. Despite several improvements minimizing radiation scatter to surrounding tissues, there is a definitely increased risk for certain malignancies following external beam radiation for breast cancer [40]. While some reports are conflicting [41], most available data do not substantiate such a connection between thyroid cancer and previous adjuvant breast irradiation [42–45]. Similarly, radioactive iodine treatment given for thyroid cancer does not seem to play a role in subsequent breast cancer development [46–48]. Our data with similar frequency of radiation therapy for breast cancer in the investigated groups also argue against the role of external or internal radiation in the development of synchronous/metachronous breast and thyroid cancer.

The increased co-occurrence of these tumor types could also be related to surveillance bias. The higher compliance with, and higher rate of screening efforts among cancer survivors could also affect the time of diagnosis and could lead the substantial overdiagnosis of clinically irrelevant second primary malignancy [49]. This seems to be especially true for differentiated thyroid cancer [50]. However, as there are no screening programs for breast cancer among thyroid cancer survivors or vice versa, surveillance bias is unlikely to account for the preferential association of these tumor types.

Cowden syndrome (CS) and Cowden-like syndromes (CLS) are characterised by hamartomas and an extremely increased risk for several types of malignancies including breast cancer, thyroid cancer, endometrial cancer, colorectal cancer, and melanoma with standardized incidence ratios for breast and thyroid cancer in the range of 6 to 9 [14]. CS and most forms of CLS have an autosomal dominant inheritance. However, these conditions, referred to as PTEN hamartoma tumor syndromes, make up only a small fraction of synchronous/metachronous thyroid and breast cancer cases. This is consistent with our finding that no patient in our samples had a mutation in the PTEN gene. Thus, the existence of other shared genetic risk factors is highly probable. To the best of our knowledge, genetic analyses exploring this notion have not been conducted and genetic factors underlying sporadic metachronous cancer cases are yet to be elucidated.

The main strengths of this study were the state-of-the-art genetic analysis using NGS technology, and the fact that both cases and controls came from the same source population. The main limitation of our research was the relatively low number of patients limiting statistical power even in analyses of genetic scores. Furthermore, our study was unable to identify or investigate any single genetic risk locus behind synchronous/metachronous cancer development. Two types of bias could also have affected our findings. First, participants were recruited years after diagnosis potentially leading to survivor bias. Second, the exclusion of controls with less than 12 years of follow-up lead to an age difference between our study groups that is contrary to previous findings [22], and probably reflects selection bias. However, both of these selections were deemed to be necessary to minimize contamination of the control group with thyroid cancer.

## Conclusions

In conclusion, we reported an increased burden of carcinogenic SNPs in people with both thyroid and breast cancer compared to individuals with breast cancer only, based on whole exome sequencing of 112 known hereditary cancer risk genes. We found no differences in clinicopathologic parameters between the groups suggesting that both groups have similar presentation at the time of diagnosis of breast cancer. While our study was not powered to identify specific risk loci for metachronous cancer development, our findings further support the multigenetic etiology of co-occurring breast and thyroid cancer. Our findings do not directly contradict any of the other, previously detailed theories explaining the association between these tumor types. Nevertheless, our results do underline the need for further genetic research in this field, which are lacking at the moment.

## Data Availability

All data generated or analysed during this study are included in this published article.

## Abbreviations

BC: Breast cancer
BCa 95% CI: Bias-corrected and accelerated 95% bootstrap confidence interval
CLS: Cowden-like syndromes
CS: Cowden syndrome
EDC: Endocrine disrupting chemical
hCG: Human chorionic gonadotropin
IGV: Integrative genomics viewer
ISP: Ion sphere particles
SD: Standard deviation
SERM: Selective estrogen receptor modulator
TC: Thyroid cancer
TRH: Thyrotropin-releasing hormone
